# Joint association analysis of bivariate quantitative and qualitative traits

**DOI:** 10.1186/1753-6561-5-S9-S74

**Published:** 2011-11-29

**Authors:** Mengdie Yuan, Guoqing Diao

**Affiliations:** 1 Department of Statistics, George Mason University, MS 4A7, 4400 University Drive, Fairfax, VA 22030, USA

## Abstract

Univariate genome-wide association analysis of quantitative and qualitative traits has been investigated extensively in the literature. In the presence of correlated phenotypes, it is more intuitive to analyze all phenotypes simultaneously. We describe an efficient likelihood-based approach for the joint association analysis of quantitative and qualitative traits in unrelated individuals. We assume a probit model for the qualitative trait, under which an unobserved latent variable and a prespecified threshold determine the value of the qualitative trait. To jointly model the quantitative and qualitative traits, we assume that the quantitative trait and the latent variable follow a bivariate normal distribution. The latent variable is allowed to be correlated with the quantitative phenotype. Simultaneous modeling of the quantitative and qualitative traits allows us to make more precise inference on the pleiotropic genetic effects. We derive likelihood ratio tests for the testing of genetic effects. An application to the Genetic Analysis Workshop 17 data is provided. The new method yields reasonable power and meaningful results for the joint association analysis of the quantitative trait Q1 and the qualitative trait disease status at SNPs with not too small MAF.

## Background

Statistical methods for the univariate association analysis of quantitative and qualitative traits have been well developed in the literature. Complex human diseases are often characterized by multiple traits. These traits tend to be correlated with each other because of common environmental and genetic factors. In the genetic analysis of complex diseases, it is natural to account for the correlations among multiple traits and to model them simultaneously. Joint genetic linkage analysis of multiple correlated phenotypes has been studied by Jiang and Zeng [1], Mangin et al. [2], Amos and Laing [3], Almasy et al. [4], Blangero et al. [5], Wijsman and Amos [6], and Williams et al. [7,8], among others. Joint linkage analysis of multiple correlated traits can potentially improve the power to detect linkage signals at genes that jointly influence a complex disease.

Recently, Liu et al. [9] developed an extended generalized estimating equation method for the bivariate association analysis of continuous and binary traits. Their simulation results demonstrated that, compared with univariate analysis, bivariate analysis may substantially improve power while having comparable type I error rates under certain situations.

In this paper we extend the joint linkage analysis of multivariate qualitative and quantitative traits described by Williams et al. [7,8] to association analysis. Specifically, we assume that a latent variable determines the qualitative trait and that the latent variable and the quantitative trait follow a bivariate normal distribution. With such modeling, we develop likelihood-based inference procedures for testing pleiotropic genetic effects. As an illustration, we perform the joint association analysis of the quantitative trait Q1 and the qualitative trait disease status on chromosome 13 from the Genetic Analysis Workshop 17 (GAW17) data.

## Methods

Suppose that the data contain *n* independent individuals. Let *Y_i_*_1_ be the qualitative trait and *Y_i_*_2_ be the quantitative trait for the *i*th individual. Let **X***_i_* denote a vector of covariates, including the intercept and environmental variables, and let **Z***_i_* denote a vector of genotype score(s) at the major single-nucleotide polymorphism (SNP) locus. We may also include gene by environment interaction terms. We assume that *Y_i_*_1_ is determined by a latent continuous variable  such that:(1)

We consider the following model:(2)

where the *β* are regression coefficients for the environmental effects, the *γ* are regression coefficients for the genetic effects, and the subscripts 1 and 2 denote the qualitative and quantitative traits, respectively, and the  are independent and identically distributed bivariate normal random variables with mean 0 and variance-covariance matrix Σ:(3)

where σ_1_^2^ is the variance of ε_i1_, σ_2_^2^ is the variance of ε_i2_, and ρ is the correlation between ε_i1_ and ε_i2_.

To ensure the identifiability of the model, we fix *σ*_1_ = 1 and *α* = 0.

Given *Y_i_*_2_, it can be shown that  follows a normal distribution with mean(4)

and variance:(5)

Therefore the likelihood function for the unknown parameters *θ* ≡ (*β*_1_, *γ*_1_, *β*_2_, *γ*_2_, *σ*_2_, *ρ*) takes the form:(6)

where:(7)

and Φ is the cumulative distribution function of the standard normal distribution.

To obtain the maximum-likelihood estimator (MLE) of *θ*, denoted , we maximize the log-likelihood by using the quasi-Newton algorithm described by Press et al. [10]. We can perform various association analyses through the likelihood ratio test (LRT) statistic, given by:(8)

where  is the MLE of *θ* under the null hypothesis. The LRT follows a chi-square distribution asymptotically with the degrees of freedom being the difference of the number of free parameters under the null hypothesis and the number under the alternative hypothesis.

## Results

**Figure 1:**
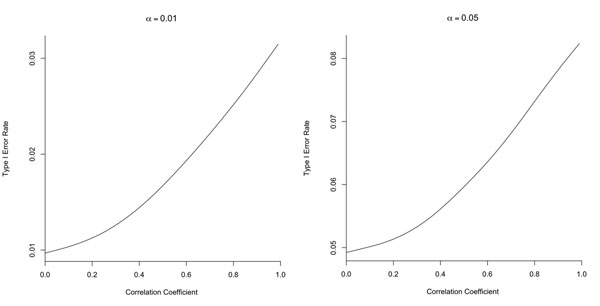
Type I error rates of the univariate association test of pleiotropic effects at the significance levels of 0.01 and 0.05.

We first conducted a small simulation study to evaluate the type I error rates of the association test based on the univariate analysis at significance levels of 0.01 and 0.05. Figure 1 presents the results based on 100,000 replicates, where the *x*-axis denotes the correlation between the two Wald test statistics for the two traits. Figure 1 implies that failing to account for the correlation between traits may lead to inflated type I error rate.

We next performed a joint association analysis of the quantitative trait Q1 and the qualitative trait disease status from the GAW17 data. Before conducting the analyses, we had knowledge of the answers. Sex, Age, and Smoking were included as covariates in the model. We evaluated the additive effect of each of the 11 true SNPs on chromosome 13 on each individual trait as well as the pleiotropic effect on both traits. The genotype score was coded as the number of minor alleles at each SNP locus. For comparison, we also performed a univariate association analysis and used the sum of the LRT statistics for testing genetic effect on an individual trait as the test statistic for testing the pleiotropic effect.

Table 1 presents the powers of the tests at the 11 true SNPs on chromosome 13 from the joint association analysis and the powers from the univariate analysis based on 200 replicates. Table 2 presents the type I error rates at 6 randomly chosen SNPs on chromosome 13, excluding the 11 true SNPs. None of these 6 SNPs was in linkage disequilibrium with the 11 true SNPs on chromosome 13. Type I error rates and powers of the association tests appear to depend very much on the minor allele frequency (MAF). Type I error rates of both tests are lower than the nominal significance level when the MAF is small. Essentially no tests have power to detect the SNP effect when the MAF is low and the minor allele is detected in only a few of the 697 individuals. On the other hand, both methods had good power to detect the pleiotropic effects at true SNPs with MAF > 0.0043. The estimates of the additive effects and the correlations for the 11 true SNPs are shown in Table 3. As expected, a strong correlation exists between quantitative trait Q1 and disease status with an estimated correlation of 0.68.

**Table 1:** Power of the association tests at a significance level of 5%

SNP	MAF	Joint analysis			Univariate analysis		
	
		*H*_0_: *γ*_1_ = 0	*H*_0_: *γ*_2_ = 0	*H*_0_: *γ*_1_ = *γ*_2_ = 0	*H*_0_: *γ*_1_ = 0	*H*_0_: *γ*_2_ = 0	*H*_0_: *γ*_1_ = *γ*_2_ = 0
C13S399	0.0007	0.045	0.020	0.010	0.060	0.020	0.030
C13S479	0.0007	0.000	0.045	0.025	0.040	0.045	0.110
C13S505	0.0007	0.030	0.025	0.015	0.030	0.025	0.040
C13S514	0.0007	0.000	0.045	0.010	0.000	0.045	0.065
C13S547	0.0007	0.050	0.020	0.050	0.075	0.020	0.050
C13S567	0.0007	0.000	0.025	0.010	0.000	0.025	0.015
C13S320	0.0014	0.020	0.025	0.010	0.005	0.025	0.050
C13S524	0.0043	0.330	1.000	1.000	0.335	1.000	1.000
C13S431	0.0172	0.210	1.000	0.995	0.185	1.000	0.985
C13S522	0.0279	0.770	1.000	1.000	0.760	1.000	1.000
C13S523	0.0667	0.960	1.000	1.000	0.965	1.000	1.000

*γ*_1_ is the additive SNP effect on the qualitative trait. *γ*_2_ is the additive SNP effect on the quantitative trait.

**Table 2:** Type I error rate of the association tests at a significance level of 5%

SNP	MAF	Joint analysis			Univariate analysis		
	
		*H*_0_: *γ*_1_ = 0	*H*_0_: *γ*_2_ = 0	*H*_0_: *γ*_1_ = *γ*_2_ = 0	*H*_0_: *γ*_1_ = 0	*H*_0_: *γ*_2_ = 0	*H*_0_: *γ*_1_ = *γ*_2_ = 0
C13S722	0.0007	0.000	0.030	0.010	0.000	0.030	0.025
C13S202	0.0022	0.005	0.065	0.055	0.005	0.065	0.055
C13S556	0.0022	0.035	0.020	0.025	0.050	0.020	0.025
C13S1580	0.0022	0.035	0.025	0.015	0.040	0.025	0.025
C13S398	0.0122	0.050	0.050	0.050	0.050	0.050	0.060
C13S1004	0.0122	0.050	0.030	0.060	0.050	0.030	0.050

*γ*_1_ is the additive SNP effect on the qualitative trait. *γ*_2_ is the additive SNP effect on the quantitative trait.

**Table 3:** Estimate of the additive effects and the correlation for the 11 true SNPs on chromosome 13

SNP	Joint analysis			Univariate analysis	
	
	*γ*_1_	*γ*_2_	*ρ*	*γ*_1_	*γ*_2_
C13S320	1.721	0.296	0.687	2.311	0.296
C13S399	−1.548	0.149	0.687	−2.760	0.149
C13S431	0.300	0.821	0.690	0.329	0.821
C13S479	−0.459	0.598	0.687	−1.230	0.598
C13S505	−2.283	0.065	0.687	−3.865	0.065
C13S514	0.575	0.320	0.686	0.304	0.320
C13S522	0.616	1.134	0.684	0.617	1.134
C13S523	0.586	1.017	0.679	0.597	1.017
C13S524	0.672	1.894	0.688	0.700	1.894
C13S547	−1.926	0.290	0.687	−3.332	0.290
C13S567	−0.689	−0.124	0.687	−0.826	−0.124

*γ*_1_ is the additive SNP effect on the qualitative trait. *γ*_2_ is the additive SNP effect on the quantitative trait. *ρ* is the correlation between the quantitative trait and the qualitative trait.

## Discussion and conclusions

In this paper, we described a likelihood-based approach for the joint association analysis of quantitative and qualitative traits in unrelated individuals. As expected from the true data generation model, our association analysis of the GAW17 data reveals that there is strong correlation between the quantitative trait Q1 and the qualitative trait disease status. In this case, the joint association test of pleiotropic effects is more appropriate than the test based on univariate analysis, which ignores the trait correlation.

Two factors might have contributed to the small difference between the joint analysis and the univariate analysis using the GAW17 data set. First, both methods had low power with rare variants, for example, when MAF < 0.0014. On the other hand, when the MAF was moderate and the effect size was relatively large, both methods yielded power close to or equal to 100%; therefore we are unlikely to see the difference between the two methods under such situations. In fact, it may not be fair to compare the powers between the two methods because the univariate analysis tends to inflate type I error rates in the presence of correlation between the qualitative trait and the quantitative trait.

In its current form, the single-SNP joint analysis is more ideal for common variants and less ideal for rare variants. To improve the power of the association tests with rare variants, we could apply the approaches of Morris and Zeggini [11] based on accumulations of rare variants within the same functional unit. The idea is to treat the proportion of rare variants at which an individual carries a minor allele as a covariate in the joint analysis model and to test for the covariate effects on the qualitative and quantitative traits jointly.

## Competing interests

The authors declare that there are no competing interests.

## Authors’ contributions

GD was responsible for the study design and helped to draft the manuscript. MY performed the statistical analysis and drafted the manuscript. Both authors read and approved the final manuscript.
